# Effects of increasing trypsin inhibitor on nursery pig growth performance, fecal dry matter, and nutrient digestibility

**DOI:** 10.1093/tas/txag126

**Published:** 2026-08-13

**Authors:** Sierra M Collier, Paige T Soper, Katelyn N Gaffield, Robert D Goodband, Mike D Tokach, Jason C Woodworth, Joel M DeRouchey, Hari B Krishnan, Jordan T Gebhardt

**Affiliations:** Department of Animal Sciences and Industry, College of Agriculture, Kansas State University, Manhattan, KS 66506-0201, United States; Department of Animal Sciences and Industry, College of Agriculture, Kansas State University, Manhattan, KS 66506-0201, United States; Department of Animal Sciences and Industry, College of Agriculture, Kansas State University, Manhattan, KS 66506-0201, United States; Department of Animal Sciences and Industry, College of Agriculture, Kansas State University, Manhattan, KS 66506-0201, United States; Department of Animal Sciences and Industry, College of Agriculture, Kansas State University, Manhattan, KS 66506-0201, United States; Department of Animal Sciences and Industry, College of Agriculture, Kansas State University, Manhattan, KS 66506-0201, United States; Department of Animal Sciences and Industry, College of Agriculture, Kansas State University, Manhattan, KS 66506-0201, United States; Agricultural Research Service, U.S. Department of Agriculture, and Division of Plant Science and Technology, University of Missouri, Columbia MO 65211, United States; Department of Diagnostic Medicine/Pathobiology, College of Veterinary Medicine, Kansas State University, Manhattan, KS 66506-0201, United States

**Keywords:** antinutritional factors, soybean meal, trypsin inhibitor, weanling pigs

## Abstract

A total of 360 barrows (DNA 200 × 400; initially 6.1 ± 0.03 kg) were used in a 42-d growth trial to evaluate the effects of increasing trypsin inhibitor units (TIU) in complete feed on nursery pig growth performance, fecal dry matter (DM), and nutrient digestibility. At weaning, pigs were blocked by BW and randomly assigned to pens with five pigs per pen and 12 pens per treatment. There were six dietary treatments formulated to provide 1.4, 2.1, 2.8, 3.5, 4.2, and 4.9 TIU/mg of complete feed. Soy flour (76 TIU/mg) was added at the expense of soybean meal (5.4 TIU/mg) to create the experimental diets. Diet formulation was based on analyzed nutrient values of the soy flour and soybean meal, assuming digestibility coefficients for soy flour were equivalent to those of soybean meal. Experimental diets were fed in three phases: phase 1 from d 0 to 10, phase 2 from d 10 to 24, and phase 3 from d 24 to 42 after weaning. Feces were collected from three pigs per pen on d 10 and 42 to determine fecal DM, and d 42 fecal samples were used to determine apparent total tract digestibility (ATTD) of DM and crude protein (CP). Across all three individual phases and overall (d 0 to 42), increasing TIU decreased (linear, *P* < 0.001) average daily gain (ADG), average daily feed intake (ADFI), and gain:feed ratio (G:F). Fecal DM on d 10 increased (linear, *P* < 0.015; quadratic, *P* = 0.088) as TIU increased, with the majority of increase in fecal DM observed as trypsin inhibitor increased from 3.5 to 4.9 TIU/mg of complete feed. However, there were no differences in fecal DM on d 42. The ATTD of DM decreased then increased (quadratic, *P* = 0.003) as TIU increased with the lowest ATTD of DM observed at 4.2 TIU/mg of complete feed. The ATTD of CP decreased (linear, *P* < 0.001) as TIU/mg of complete feed increased. In conclusion, these data suggest that increasing dietary TIU above 1.4 TIU/mg of complete feed linearly decreased nursery pig growth performance and reduced the ATTD of DM and CP. Based on these results, each 1 TIU/mg increase in dietary TIU was associated with a 38 g/d, or a 7.4%, reduction in overall ADG. Overall ADFI decreased by approximately 40 g/d, or 5%, per 1 TIU/mg increase. Overall G:F decreased approximately 3% per 1 TIU/mg increase. Based on the amounts of SBM used in the diets for this study, and its TIU concentrations, soybean meal with greater than 5.4 TIU/mg has the potential to decrease nursery pig performance.

## Teaser

Increasing trypsin inhibitor reduced nursery pig growth performance and nutrient digestibility, underscoring the need for close monitoring of soybean meal quality.

## Introduction

Soybean meal (SBM) is the predominant plant-based protein source used in swine diets due to its favorable amino acid profile, high digestibility, and consistent supply across global markets. However, SBM contains antinutritional factors (ANF) such as trypsin inhibitor, that if not thermally processed correctly, can reduce its nutritional value, most notably for young pigs transitioning to solid feed after weaning (Liener 1976; Vagadia et al. 2017). During this period, pigs commonly experience a transitory period of hypersensitivity to antigenic soy proteins, which can contribute to postweaning diarrhea (PWD), villus atrophy, and reduced growth performance (Li et al. 1991; Pluske et al. 1997; Lalles et al. 2004). Trypsin inhibitor decreases protein and amino acid digestibility by interfering with pancreatic protease activity of trypsin and chymotrypsin (Liener, 1976; Goebel and Stein 2010). Trypsin inhibitors occur naturally in soybeans as two major protein families known as Kunitz trypsin inhibitor (KTI) and Bowman-Birk inhibitor (BBI) that protect the plant from predation (Kunitz 1946; Birk 1985). These proteins bind with trypsin and chymotrypsin, resulting in pancreatic enzyme hypersecretion, increased endogenous amino acid losses, and reduced growth performance (Schneeman et al. 1977; Gu et al. 2010). Weanling pigs have limited digestive capacity, so even modest concentrations of TIU can elicit disproportionate reductions in protein utilization and feed efficiency (Zhang et al. 2020).

Despite improvements in commercial soy processing, a recent survey observed substantial variation in TIU concentrations among SBM sources in the United States (Gaffield et al. 2024). The results of this industry survey of over 14 soy processing plants indicated wide variations in TIU across SBM sources in the United States, with values ranging from 1.67 to 10.57 TIU/mg. This variation in soybean meal TIU has prompted the resurgence of research evaluating the acceptable levels of TIU in nursery pig diets. Nisley et al. (2025) observed that increasing dietary TIU (0.50 to 6.0 TIU/mg) through the inclusion of raw soybeans linearly decreased nursery pig ADG and G:F. Similarly, Miller et al. (2025a) reported reduced nutrient digestibility and growth performance in nursery pigs when TIU was increased (0.61 to 3.51 TIU/mg) using soy flour, and Miller et al. (2025b) observed comparable reductions in growing pigs when TIU was increased (0.99 to 9.38 TIU/mg) using raw soybeans. Collectively, these studies demonstrated that elevated dietary TIU negatively affects pig performance and additional research is needed to establish practical TIU thresholds in conventional corn-soybean meal diets. Given the considerable variation in TIU observed among commercially available SBM sources, additional research is warranted to establish practical TIU thresholds using a soy flour titration model that allowed dietary TIU to be systematically increased while minimizing changes in ingredient composition beyond the increase in TIU itself.

Therefore, the objective of this study was to evaluate the effect of increasing TIU on nursery pig growth performance, fecal DM, and nutrient digestibility. While it was hypothesized that increasing dietary TIU would decrease these criteria, it was anticipated that a maximum TIU value could be established beyond which these variables would decrease.

## Materials and methods

The Kansas State University Institutional Animal Care and Use Committee approved the protocol used in this experiment (IACUC #4943). This study was conducted at the Kansas State University Segregated Early Weaning facility in Manhattan, KS. The facility has two identical barns that are completely enclosed, environmentally controlled, and mechanically ventilated. Each pen (1.2 × 1.2 m) contained a 4-hole, dry self-feeder and a cup waterer providing ad libitum access to feed and water. Facilities were mechanically ventilated with room temperatures set at 32.2°C during the first 10 d and then gradually reduced by 0.28°C daily until the set point reached 25°C. Overall pig health was excellent and only 1 pig was removed from the study, not treatment related.

### Animals and diets

A total of 360 weanling barrows (DNA 200 × 400, Columbus, NE; initially 6.1 ± 0.03 kg) were used across 2 barns in a 42-d growth trial. Pigs were weaned at approximately 21 d of age and assigned to pens blocked by initial body weight (BW). Treatments were assigned in a generalized randomized block design. Pigs were blocked into light (initially 5.1 ± 0.02 kg), intermediate (initially 6.0 ± 0.02 kg), and heavy groups (initially 7.1 ± 0.02 kg). Within each block, there were 4 pens of 5 pigs per treatment (2 pens per weight group in each barn) for a total of 12 replications per treatment (six per barn). Pens of pigs were randomly allotted to one of six dietary treatments. Diets were corn-soybean meal-based and consisted of increasing TIU/mg of complete feed (1.4, 2.1, 2.8, 3.5, 4.2, or 4.9). The lowest TIU containing diet was based on what might be considered common U.S. industry soybean meal inclusion levels for nursery pig diets. Soy flour (Prolia FLR-100/90760, Cargill, Minneapolis, MN: 76 TIU/mg) was added at the expense of soybean meal (5.4 TIU/mg) to achieve increasing TIU levels while having the same total soy product inclusion. Dietary TIU were formulated to be identical across the three phases. Samples of soybean meal and soy flour were collected prior to diet formulation and analyzed at the University of Missouri Agricultural Experiment Station Chemical Laboratory for dry matter (Method 934.01; AOAC 2006), crude protein (Method 990.03; AOAC 2006), crude fat (Method 920.39; AOAC 2002), crude fiber (Method 978.10, AOAC 2006), ash (Method 942.05; AOAC 2005), and complete AA profile (Method 982.30 E (a, b, c), 988.15; AOAC 2006), and protein dispersibility index (AOCS 2017). The soybean meal and soy flour were analyzed in triplicate for TIU at Agricultural Research Service, U.S. Department of Agriculture (Columbia, MO) following the procedure described by Kim and Krishnan (2023; Table 1). Diets were then formulated using these analyzed nutrient values of the soy flour and soybean meal, assuming digestibility coefficients for soy flour equivalent to those of soybean meal (NRC 2012). The net energy of soy flour was assumed to be the same as soybean meal.

**Table 1:** Chemical analysis of soybean meal and soy flour (as-fed basis)^a^^,^^b^.

Item	Soybean meal	Soy flour
**Trypsin inhibitor units, TIU/mg**	5.41	75.87
**Proximate analysis, %**		
** Dry matter**	91.92	93.04
** Crude protein**	48.45	50.18
** Crude fat**	3.00	0.62
** Crude fiber**	3.09	3.97
** Ash**	6.05	6.02
** Protein dispersibility index**	10.27	76.48
**Total essential amino acid concentration, %**		
** Cys**	0.71	0.74
** His**	1.30	1.33
** Ile**	2.28	2.33
** Leu**	3.69	3.79
** Lys**	3.21	3.27
** Met**	0.73	0.68
** Thr**	1.92	1.95
** Trp**	0.58	0.59
** Val**	2.38	2.40

a Values represent the mean of two samples analyzed in duplicate.

b Ingredient samples were submitted for complete proximate and amino acid analysis in duplicate (University of Missouri Agricultural Experiment Station Chemical Laboratory; Columbia, MO). Protein dispersibility index was determined at the Kansas State University (Manhattan, KS) Swine Laboratory. Trypsin inhibitor units were analyzed in triplicate at the Agricultural Research Service, U.S. Department of Agriculture (Columbia, MO). Analyzed values were used in diet formulation.

Pigs were fed treatment diets in meal form for all three nursery phases with phase 1 from d 0 to 10, phase 2 from d 10 to 24, and phase 3 from d 24 to 42 (Tables 2 to 4). Diets were fed in mash form to eliminate any potential TIU denaturation from the pelleting process. Treatment diets were manufactured at the Kansas State University O.H. Kruse Feed Technology Innovation Center in Manhattan, KS. During bagging, complete diet samples were collected from every third bag using a feed probe, pooled, and stored at −20°C until analysis. Diet samples were analyzed at the Kansas State University Swine Laboratory for DM and CP, while TIU was analyzed similar to the ingredient analysis of soybean meal and soy flour (Tables 2 to 4).

**Table 2:** Phase 1 diet composition (as-fed basis)^a^.

Item	Trypsin inhibitor units (TIU), per mg of complete feed					
	1.4	2.1	2.8	3.5	4.2	4.9
**Ingredients, %**						
** Corn**	51.26	51.28	51.30	51.28	51.27	51.29
** Soybean meal**	17.57	16.55	15.53	14.55	13.56	12.54
** Soy flour**	0.55	1.55	2.55	3.55	4.55	5.55
** Whey powder**	12.50	12.50	12.50	12.50	12.50	12.50
** Whey permeate, 80% lactose**	7.50	7.50	7.50	7.50	7.50	7.50
** Spray-dried blood plasma**	2.50	2.50	2.50	2.50	2.50	2.50
** Fish meal**	5.00	5.00	5.00	5.00	5.00	5.00
** Calcium carbonate**	0.53	0.53	0.53	0.53	0.53	0.53
** Monocalcium, 21% P**	0.45	0.45	0.45	0.45	0.45	0.45
** Salt**	0.40	0.40	0.40	0.40	0.40	0.40
** L-Lys-HCl**	0.34	0.34	0.34	0.34	0.34	0.34
** DL-Met**	0.19	0.19	0.19	0.19	0.19	0.19
** L-Thr**	0.18	0.18	0.18	0.18	0.18	0.18
** L-Trp**	0.05	0.05	0.05	0.05	0.05	0.05
** L-Val**	0.11	0.11	0.11	0.11	0.11	0.11
** L-Ile**	0.03	0.03	0.03	0.03	0.03	0.03
** Zinc oxide**	0.40	0.40	0.40	0.40	0.40	0.40
** Vitamin premix^b^**	0.25	0.25	0.25	0.25	0.25	0.25
** Trace mineral premix^c^**	0.15	0.15	0.15	0.15	0.15	0.15
** Phytase^d^**	0.06	0.06	0.06	0.06	0.06	0.06
** Total**	100	100	100	100	100	100
**Calculated analysis**						
**Standardized ileal digestible amino acids, %**						
** Lys**	1.35	1.35	1.35	1.35	1.35	1.35
** Ile:Lys**	56	56	56	56	56	56
** Leu:Lys**	112	112	112	112	112	113
** Met:Lys**	37	37	37	36	36	36
** Met and Cys:Lys**	58	58	58	58	58	58
** Thr:Lys**	64	64	64	64	64	64
** Trp:Lys**	19.2	19.2	19.2	19.2	19.2	19.2
** Val:Lys**	70	70	70	70	70	70
** His:Lys**	34	34	34	34	34	34
** Net energy, kcal/kg**	2,518	2,518	2,518	2,518	2,518	2,518
** SID Lys:net energy, g/Mcal**	5.36	5.36	5.36	5.36	5.36	5.36
** Crude protein, %**	20.5	20.5	20.5	20.5	20.5	20.6
** STTD P, %**	0.57	0.57	0.57	0.57	0.57	0.57
**Analyzed composition**						
** TIU/mg of complete feed**	1.86	2.84	3.48	4.52	4.59	5.29
** Crude protein, %**	18.8	18.1	17.1	20.0	18.7	19.3
** Dry matter, %**	89.0	89.1	89.1	89.0	89.1	89.2

a Diets were fed from d 0 to 10.

b Provided per kg of feed: 4134 IU of vitamin A as retinyl acetate; 1653 IU of vitamin D_3_ as cholecalciferol; 44 IU vitamin E as dl-α-tocopherol acetate; 3 mg of vitamin K as menadione; 0.03 mg of vitamin B_12_ as cyanocobalamin; 50 mg of niacin as niacinamide; 26 mg of pantothenic acid as d-calcium pantothenate; 8 mg of riboflavin as crystalline riboflavin.

c Provided per kg of feed: 17 mg of Cu as copper sulfate; 0.3 mg I as ethylenediamine dihydriodide; 110 mg Fe as ferrous sulfate; 33 mg Mn as manganese oxide; 0.3 mg Se as sodium selenite; 110 mg of Zn as zinc sulfate.

d HiPhorius 2400 (dsm-firmenich, Plainsboro, NJ) was added at 1,500 FYT/kg feed and provided an estimated release of 0.13% STTD P.

STTD, standard total tract digestibility.

**Table 3:** Phase 2 diet composition (as-fed basis)^a^.

Item	Trypsin inhibitor units (TIU), per mg of complete feed					
	1.4	2.1	2.8	3.5	4.2	4.9
**Ingredients, %**						
** Corn**	58.45	58.47	58.45	58.44	58.46	58.48
** Soybean meal**	24.50	23.48	22.51	21.52	20.50	19.48
** Soy flour**	0.05	1.05	2.05	3.05	4.05	5.05
** Whey powder**	5.00	5.00	5.00	5.00	5.00	5.00
** Whey permeate, 80% lactose**	5.00	5.00	5.00	5.00	5.00	5.00
** Fish meal**	3.25	3.25	3.25	3.25	3.25	3.25
** Calcium carbonate**	0.63	0.63	0.63	0.63	0.63	0.63
** Monocalcium P, 21% P**	0.75	0.75	0.75	0.75	0.75	0.75
** Salt**	0.50	0.50	0.50	0.50	0.50	0.50
** L-Lys-HCl**	0.46	0.46	0.46	0.46	0.46	0.46
** DL-Met**	0.22	0.22	0.22	0.22	0.22	0.22
** L-Thr**	0.23	0.23	0.23	0.23	0.23	0.23
** L-Trp**	0.07	0.07	0.07	0.07	0.07	0.07
** L-Val**	0.15	0.15	0.15	0.15	0.15	0.15
** L-Ile**	0.04	0.04	0.04	0.04	0.04	0.04
** Zinc oxide**	0.25	0.25	0.25	0.25	0.25	0.25
** Vitamin premix^b^**	0.25	0.25	0.25	0.25	0.25	0.25
** Trace mineral premix^c^**	0.15	0.15	0.15	0.15	0.15	0.15
** Phytase^d^**	0.06	0.06	0.06	0.06	0.06	0.06
** Total**	100	100	100	100	100	100
**Calculated analysis**						
**Standardized ileal digestible amino acids, %**						
** Lys**	1.35	1.35	1.35	1.35	1.35	1.35
** Ile:Lys**	57	57	57	57	57	57
** Leu:Lys**	109	109	109	109	109	109
** Met:Lys**	39	39	39	39	39	39
** Met and Cys:Lys**	58	58	58	58	58	58
** Thr:Lys**	64	64	64	64	64	64
** Trp:Lys**	19.1	19.1	19.1	19.1	19.1	19.1
** Val:Lys**	70	70	70	70	70	70
** His:Lys**	34	34	34	34	34	34
** Net energy, kcal/kg**	2,475	2,475	2,475	2,475	2,475	2,475
** SID Lys:net energy, g/Mcal**	5.46	5.46	5.46	5.46	5.46	5.46
** Crude protein, %**	20.4	20.4	20.4	20.5	20.5	20.5
** STTD P, %**	0.53	0.53	0.53	0.53	0.53	0.53
**Analyzed composition**						
** TIU/mg of complete feed**	1.99	2.65	3.77	4.40	4.63	4.90
** Crude protein, %**	19.1	19.1	19.0	18.9	18.8	19.2
** Dry matter, %**	88.2	87.9	88.1	87.8	87.7	87.7

a Diets were fed from d 10 to 24.

b Provided per kg of feed: 4134 IU of vitamin A as retinyl acetate; 1653 IU of vitamin D_3_ as cholecalciferol; 44 IU vitamin E as dl-α-tocopherol acetate; 3 mg of vitamin K as menadione; 0.03 mg of vitamin B_12_ as cyanocobalamin; 50 mg of niacin as niacinamide; 26 mg of pantothenic acid as d-calcium pantothenate; 8 mg of riboflavin as crystalline riboflavin.

c Provided per kg of feed: 17 mg of Cu as copper sulfate; 0.3 mg I as ethylenediamine dihydriodide; 110 mg Fe as ferrous sulfate; 33 mg Mn as manganese oxide; 0.3 mg Se as sodium selenite; 110 mg of Zn as zinc sulfate.

d HiPhorius 2400 (dsm-firmenich, Plainsboro, NJ) was added at 1500 FYT/kg feed and provided an estimated release of 0.13% STTD P.

STTD, standard total tract digestibility.

**Table 4:** Phase 3 diet composition (as-fed basis)^a^.

Item	Trypsin inhibitor units (TIU), per mg of complete feed					
	1.4	2.1	2.8	3.5	4.2	4.9
**Ingredients, %**						
** Corn**	69.54	69.53	69.51	69.53	69.54	69.54
** Soybean meal**	25.83	24.84	23.86	22.84	21.84	20.83
** Soy flour**	—	1.00	2.00	3.00	4.00	5.00
** Calcium carbonate**	0.80	0.80	0.80	0.80	0.80	0.80
** Monocalcium P, 21% P**	0.95	0.95	0.95	0.95	0.95	0.95
** Salt**	0.60	0.60	0.60	0.60	0.60	0.60
** L-Lys-HCl**	0.55	0.55	0.55	0.55	0.55	0.55
** DL-Met**	0.23	0.23	0.23	0.23	0.23	0.23
** L-Thr**	0.26	0.26	0.26	0.26	0.26	0.26
** L-Trp**	0.08	0.08	0.08	0.08	0.08	0.08
** L-Val**	0.16	0.16	0.16	0.16	0.16	0.16
** L-Ile**	0.04	0.04	0.04	0.04	0.04	0.04
** Vitamin premix^b^**	0.25	0.25	0.25	0.25	0.25	0.25
** Trace mineral premix^c^**	0.15	0.15	0.15	0.15	0.15	0.15
** Phytase^d^**	0.06	0.06	0.06	0.06	0.06	0.06
** Titanium dioxide^e^**	0.50	0.50	0.50	0.50	0.50	0.50
** Total**	100	100	100	100	100	100
**Calculated analysis**						
** Standardized ileal digestible amino acids, %**						
** Lys**	1.30	1.30	1.30	1.30	1.30	1.30
** Ile:Lys**	56	56	56	56	56	56
** Leu:Lys**	109	109	109	109	109	110
** Met:Lys**	38	38	38	38	38	38
** Met and Cys:Lys**	58	58	58	58	58	58
** Thr:Lys**	64	64	64	64	64	64
** Trp:Lys**	19.1	19.1	19.1	19.1	19.1	19.1
** Val:Lys**	70	70	70	70	70	70
** His:Lys**	34	34	34	34	34	34
** Net energy, kcal/kg**	2,445	2,445	2,445	2,445	2,445	2,445
** SID Lys:net energy, g/Mcal**	5.32	5.32	5.32	5.32	5.32	5.32
** Crude protein, %**	19.3	19.3	19.3	19.3	19.4	19.4
** STTD P, %**	0.45	0.45	0.45	0.45	0.45	0.45
**Analyzed composition**						
** TIU/mg of complete feed**	1.74	3.07	4.01	4.70	5.21	5.19
** Crude protein, %**	18.2	18.4	18.0	18.2	18.7	18.3
** Dry matter, %**	86.9	86.9	87.0	87.2	87.0	87.0

a Diets were fed from d 24 to 42.

b Provided per kg of feed: 4134 IU of vitamin A as retinyl acetate; 1653 IU of vitamin D_3_ as cholecalciferol; 44 IU vitamin E as dl-α-tocopherol acetate; 3 mg of vitamin K as menadione; 0.03 mg of vitamin B_12_ as cyanocobalamin; 50 mg of niacin as niacinamide; 26 mg of pantothenic acid as d-calcium pantothenate; 8 mg of riboflavin as crystalline riboflavin.

c Provided per kg of feed: 17 mg of Cu as copper sulfate; 0.3 mg I as ethylenediamine dihydriodide; 110 mg Fe as ferrous sulfate; 33 mg Mn as manganese oxide; 0.3 mg Se as sodium selenite; 110 mg of Zn as zinc sulfate.

d HiPhorius 2400 (dsm-firmenich, Plainsboro, NJ) was added at 1500 FYT/kg feed and provided an estimated release of 0.13% STTD P.

e Utilized as an indigestible marker for apparent total tract digestibility calculations.

STTD, standard total tract digestibility.

Pig weights and feed disappearance were measured on d 0, 10, 17, 24, 31, and 42 to determine average daily gain (ADG), average daily feed intake (ADFI), and gain-to-feed ratio (G:F).

### Dry matter and digestibility calculations

Feces were collected from 3 pigs per pen on d 10 and 42 to determine percentage fecal dry matter (DM). Fecal samples were analyzed separately for each pig, and the average of the three samples from each pen was then used for statistical analysis. Fecal samples were dried at 55°C for 48 h. The loss of weight was used to calculate percentage fecal DM. Additionally, titanium dioxide (0.50%) was added in phase 3 diets as an indigestible marker to determine apparent total tract digestibility (ATTD) of DM and CP from samples collected on d 42. Following fecal DM determination, both ground feed and fecal samples were dried at 135°C for 2 h to determine percentage DM of the samples used for titanium analysis (Method 985.01; AOAC 2007). Titanium dioxide concentration in both dried feed and fecal samples was determined utilizing procedures outlined by Leone (1973). The ATTD of DM and CP were determined using the index method described by Adeola (2001).

### Statistical analysis

Data were analyzed as a generalized randomized block design for one-way ANOVA using the lmer function from the lme4 package in R Studio (version 3.5.2, R Core team. Vienna, Austria). Pen served as the experimental unit, dietary treatment and weight block served as fixed effects, and barn was included as a random effect. Linear and quadratic contrasts were used to test for increasing TIU. Contrast statements for fecal DM were used to test the main effects of treatment, day, and their interaction. Results were considered significant with *P* < 0.05 and marginally significant at 0.05 < *P* ≤ 0.10.

## Results

Analysis of final diets for TIU/mg of complete feed were generally higher compared to diet formulation; however, the expected overall increase in TIU was observed for the six dietary treatments (Tables 2 to 4).

In all three dietary phases and overall (d 0 to 42), increasing dietary TIU decreased (linear, *P* < 0.001) BW, ADG, ADFI, and G:F (Table 5). From d 0 to 10, ADG was decreased by 36% and G:F by 22% for pigs fed 4.9 TIU/mg of complete feed compared with those fed 1.4 TIU/mg. On d 42, pigs fed 4.9 TIU/mg of complete feed were 5.6 kg lighter than those fed 1.4 TIU/mg. The decrease in ADG was partially driven by a reduction in ADFI (linear, *P* < 0.001) as TIU increased, during each phase and overall.

**Table 5:** Effects of increasing trypsin inhibitor units on nursery pig growth performance fecal dry matter, and nutrient digestibility^a^.

Item	Trypsin inhibitor units (TIU), per mg of complete feed^b^						SEM	*P* =	
	1.4	2.1	2.8	3.5	4.2	4.9		Linear	Quadratic
**BW, kg**									
** d 0**	6.1	6.1	6.1	6.1	6.1	6.1	0.03	0.926	0.620
** d 10**	8.5	8.3	8.2	8.1	7.9	7.6	0.10	<0.001	0.681
** d 24**	15.0	14.4	14.0	13.4	13.1	12.3	0.19	<0.001	0.622
** d 42**	27.8	27.1	26.0	24.6	23.2	22.2	0.35	<0.001	0.401
**Phase 1 (d 0 to 10)**									
** ADG, g**	246	220	208	200	185	157	8.1	<0.001	0.773
** ADFI, g**	313	296	293	290	289	256	11.7	<0.001	0.394
** G:F, g/kg**	787	738	710	692	638	613	17.6	<0.001	0.937
**Phase 2 (d 10 to 24)**									
** ADG, g**	465	437	419	385	367	333	10.5	<0.001	0.728
** ADFI, g**	636	608	595	556	536	505	13.4	<0.001	0.712
** G:F, g/kg**	730	719	707	694	687	661	11.4	<0.001	0.602
**Phase 3 (d 24 to 42)**									
** ADG, g**	708	693	663	621	564	553	11.5	<0.001	0.383
** ADFI, g**	1,133	1,108	1,095	1,041	967	943	17.5	<0.001	0.144
** G:F, g/kg**	625	626	607	598	583	587	7.4	<0.001	0.600
**Overall (d 0 to 42)**									
** ADG, g**	517	491	474	442	408	385	8.1	<0.001	0.488
** ADFI, g**	772	741	737	700	662	633	11.4	<0.001	0.236
** G:F, g/kg**	669	662	644	632	617	609	5.9	<0.001	0.870
**Fecal dry matter, %^c^**									
** d 10**	25.87	25.42	25.44	25.83	26.80	27.59	0.637	0.015	0.088
** d 42**	27.46	26.87	26.15	27.65	26.36	27.54	0.637	0.943	0.261
**Apparent total tract digestibility, %**									
** Dry matter**	85.65	84.27	84.69	83.26	80.74	84.88	0.647	0.001	0.003
** Crude protein**	79.87	77.19	77.55	75.22	75.22	75.55	0.952	<0.001	0.105

a A total of 360 barrows (Line 200 × 400, DNA, Columbus NE, initially 6.1 ± 0.03 kg) were used with five pigs per pen and 12 replicates per treatment.

b Soy flour and soybean meal were blended to create the TIU/mg of complete feed for dietary treatments.

c Feces from three pigs per pen were weighed and dried to measure fecal dry matter. Linear treatment × day interaction, *P* = 0.094; Treatment, *P* = 0.120; Day, *P* = 0.023.

There was a tendency for an interaction (linear, *P* = 0.094) between treatment and day for fecal DM. On d 10, fecal DM increased (linear, *P* < 0.015; quadratic, *P* = 0.088) as TIU/mg of complete feed increased, with the majority of the increase in fecal DM observed as TIU increased from 3.5 to 4.9 TIU/mg. However, there were no differences in fecal DM on d 42. Additionally, there was a main effect of day (*P* < 0.05) with increased fecal DM on d 42 compared with d 10.

The ATTD of DM decreased (quadratic, *P* = 0.003) as TIU of complete feed increased from 1.4 to 4.2 TIU/mg and then increased at 4.9 TIU/mg to values similar to those observed for pigs fed 2.8 TIU/mg. The ATTD of CP decreased (linear, *P* < 0.001) as the level of TIU/mg of complete feed increased in the diet.

## Discussion

The amount of SBM in nursery diets is often limited due to ANFs that can be related to decreased nursery growth performance, which can ultimately affect profitability for producers. In this study, increasing dietary TIU concentration had a negative effect on growth performance as a result of poorer feed intake, feed efficiency, and nutrient digestibility than pigs fed the control diet. These findings are relevant given the substantial variation in TIU levels observed across U.S. soybean meal sources in an industry survey (Gaffield et al. 2024). This wide range in SBM quality means that nursery diets may unknowingly contain high TIU levels, which is especially problematic for newly weaned pigs with limited digestive enzymatic functions that are most susceptible to ANFs. Even slight increases in dietary TIU can disproportionately decrease digestibility, nutrient utilization, and growth performance, prompting the need to determine the concentration at which performance begins to decline.

The magnitude and linear responses observed herein are comparable with recent nursery pig studies. Nisley et al. (2025) and Miller et al. (2025a) both reported linear reductions in ADG, ADFI, and G:F using ground raw soybeans and soy flour to increase dietary TIU from approximately 0.5 to 6.0 and 0.61 to 3.51 TIU/mg, respectively. When combining the results of these prior studies with the current study, each 1 TIU/mg increase in dietary TIU was associated with a 37 to 39 g/d, or a 7% to 9% reduction in overall ADG. Overall ADFI responses were also consistent, decreasing by approximately 30 to 40 g/d, or 5% to 7%, per 1 TIU/mg increase. Overall G:F decreased across all three studies, with reductions ranging from approximately 3% to 6% per 1 TIU/mg increase. Importantly, dietary TIU was measured using the same assay and laboratory across all three studies, supporting direct comparison of TIU concentrations and biological responses.

In grow-finish pigs, Miller et al. (2025b) observed linear reductions in growth performance and nitrogen digestibility as dietary TIU increased from 0.99 to 9.38 TIU/mg, with final BW reduced by more than 9% and nitrogen digestibility reduced by approximately 7% at the greatest TIU concentration. The biological trajectory of these findings is remarkably similar to those in nursery pigs, in that increasing dietary TIU consistently decreases feed intake, nutrient digestibility, and growth.

In soybean processing, raw beans are dehulled, cracked, flaked, and solvent extracted to remove oil, resulting in defatted soy flakes. These soy flakes are then ground to produce soy flour or further heat processed to produce SBM. In the present study, soy flour was used as a substitute for SBM to increase dietary TIU while maintaining a constant amount of soy protein across treatments. The chemical composition of soy flour (crude protein and amino acids) analyzed similarly to soybean meal. However, soy flour has a high TIU content due to limited heat processing. This is expected because trypsin inhibitor inactivation is strongly influenced by processing conditions, including heat exposure, moisture, and treatment duration (Vagadia et al. 2017). Furthermore, soy flour was included at relatively low concentrations to achieve the targeted TIU concentrations, minimizing potential confounding effects on diet palatability (Arndt et al. 1999) and isolating differences among diets to TIU activity.

The observed increase in d 10 fecal DM as dietary TIU increased may be partially driven by reduced feed intake, as lower ADFI can decrease digesta passage rate and increase gastrointestinal retention time (Wilfart et al. 2007), potentially allowing for greater water absorption in the hindgut and result in firmer feces. Yet the absence of fecal DM response on d 42, despite the maintained decrease in growth performance, indicates that the fecal DM might not be a sensitive indicator of the persistent negative effects of dietary TIU as nursery pigs mature. It should be noted however, that all fecal DM values were relatively high exceeding 25% on both days 10 and 42.

Apparent total tract digestibility of DM displayed a quadratic response, with the lowest values near 4.2 TIU/mg, which may represent the point at which proteolytic inhibition most severely limits nutrient availability before partial compensatory adaptation in digestive function occurs (Goebel and Stein 2010). Although fecal DM did not differ among treatments at d 42, ATTD of CP measured from the same fecal samples decreased linearly as dietary TIU increased, likely reflecting inhibition of trypsin and chymotrypsin activity and increased endogenous nitrogen losses. Miller et al. (2025b) and Nisley et al. (2025) observed similar reductions in protein digestibility when TIU concentrations exceeded 3 TIU/mg, yet the study herein shows that even values below that 3 TIU/mg have a negative effect on growth and nutrient utilization. Collectively, the present and prior data reinforce the direct link between increasing dietary TIU, enzyme inhibition, and reduced nutrient digestion.

The data herein indicate that biologically relevant reductions in nursery pig performance can occur at relatively modest dietary TIU concentrations. Growth performance declined linearly across the evaluated range of 1.4 to 4.9 TIU/mg, indicating that relatively small increases in TIU may negatively affect nursery pig growth. Rather than identifying a single universal threshold, the present findings, together with Miller et al. (2025a) and Nisley et al. (2025), demonstrate a consistent negative response to increasing dietary TIU across multiple nursery pig studies. Likewise, Royer et al. (2022) and Miller et al. (2025b) observed that increasing dietary TIU can linearly decrease growth performance and nutrient utilization in older pigs. Importantly, the consistency in response across studies utilizing different TIU sources, including raw soybeans, soy flour, and soybean meal-based titration models, suggests that the observed reductions in performance are primarily attributable to TIU activity rather than ingredient-specific effects.

Although the biological responses to increasing TIU are consistent across studies, reported values where growth performance decreases vary considerably. Part of this variation may be attributable to differences in the analytical methodologies used to quantify inhibitor activity (Sueiro et al. 2015; Kim and Krishnan 2023). Although TIU, TIA, and mg trypsin inhibited/g are frequently used throughout literature, these values are not always directly comparable. Trypsin inhibitor activity generally refers to the measured inhibitory activity, whereas the reported value may be expressed using different units depending on the analytical method employed. The American Association of Cereal Chemists International (AACCI) and American Oil Chemists’ Society (AOCS) methods are derived from the procedure developed by Kakade et al. (1974) and quantify TIA using TIU, an arbitrary unit based on the reduction in absorbance of a chromogenic substrate. These methods have undergone several refinements over time, with Hamerstrand et al. (1981) introducing a simplified single-dilution approach and Kim and Krishnan (2023) further reducing assay volume and analytical complexity while maintaining comparable estimates of inhibitor activity. In contrast, the ISO 14902 method determines TIA, expressed as mg trypsin inhibited/g of sample, rather than reporting TIU. Because the ISO and AACCI/AOCS methods differ in sample preparation, extraction conditions, assay chemistry, and calculation procedures, values generated by the two methodologies are not directly interchangeable. Sueiro et al. (2015) reported greater TIA values using an AACCI-based method modified by Hamerstrand et al. (1981) compared with the ISO method, while Liu (2021a) found that although ISO and AOCS derived values were highly correlated, direct numerical conversions remained difficult. Industry resources have proposed conversion factors to facilitate interpretation; however, these relationships are assay-specific and influenced by factors such as the source and activity of the trypsin reagent (Liu 2021b). Consequently, comparisons among studies should consider both the reported TIU concentration and the analytical methodology used to generate the value. The present study utilized an AACCI/AOCS-based assay and expressed results as TIU/mg of complete feed; therefore, comparisons with studies employing similar methodologies are likely appropriate, whereas direct comparison with ISO-derived values or thresholds should be interpreted cautiously. As interest grows in establishing biologically meaningful TIU thresholds for swine diets, greater standardization of analytical methodology and reporting units will improve interpretation across studies and facilitate development of practical quality-control guidelines for soybean meal.

In summary, increasing dietary TIU decreased growth performance throughout the entire nursery period as well as decreased nutrient digestibility. Based on these results, soybean meal with greater than 5.4 TIU/mg at an inclusion similar to that used in this study would result in TIU greater than 1.4 TIU/mg of complete feed, which will lead to a reduction in nursery growth performance and nutrient digestibility. Importantly, the 5.4 TIU/mg falls within the range of values recently reported in commercially available soybean meal sources in the U.S., indicating that nursery diets formulated without consideration of TIU concentration may unknowingly exceed biologically relevant levels.

Contribution no. 26–299-J of the Kansas Agricultural Experiment Station, Manhattan, 66506-0201.

## List of abbreviations

ADG: average daily gain
ADFI: average daily feed intake
AID: apparent ileal digestibility
ANF: antinutritional factors
ATTD: apparent total tract digestibility
BBI: Bowman–Birk inhibitor
BW: body weight
G:F: gain-to-feed
KTI: Kunitz trypsin inhibitor
PWD: postweaning diarrhea
SID: standardized ileal digestibility
SBM: soybean meal
TIA: trypsin inhibitor activity
TIU: trypsin inhibitor unit
